# Effects of growth hormone on cardiac remodeling and soleus muscle in rats with aortic stenosis-induced heart failure

**DOI:** 10.18632/oncotarget.20583

**Published:** 2017-08-24

**Authors:** Aline R.R. Lima, Luana U. Pagan, Ricardo L. Damatto, Marcelo D.M. Cezar, Camila Bonomo, Mariana J. Gomes, Paula F. Martinez, Daniele M. Guizoni, Dijon H.S. Campos, Felipe C. Damatto, Katashi Okoshi, Marina P. Okoshi

**Affiliations:** ^1^ Botucatu Medical School, Internal Medicine Departament, Sao Paulo State University, UNESP, Botucatu, Brazil; ^2^ School of Physical Therapy, Federal University of Mato Grosso do Sul, Campo Grande, Brazil

**Keywords:** heart failure, skeletal muscle, growth hormone, muscle trophicity, satellite cells

## Abstract

**Background:**

Skeletal muscle wasting is often observed in heart failure (HF). The growth hormone (GH)/insulin-like growth factor-1 (IGF-1) axis is impaired in HF. In this study, we evaluated the effects of GH on soleus muscle and cardiac remodeling in rats with aortic stenosis (AS)-induced HF.

**Methods:**

AS was created by placing a stainless-steel clip on the ascending aorta. After clinically detecting HF, GH (2 mg/kg/day) was subcutaneously injected for 14 days (AS-GH group). Results were compared with those from Sham and non-treated AS groups. Transthoracic echocardiogram was performed before and after treatment. Protein expression was evaluated by Western blot and satellite cells activation by immunofluorescence. Statistical analyzes: ANOVA and Tukey or Kruskal-Wallis and Student-Newman-Keuls.

**Results:**

Before treatment both AS groups presented a similar degree of cardiac injury. GH prevented body weight loss and attenuated systolic dysfunction. Soleus cross-sectional fiber areas were lower in both AS groups than Sham (Sham 3,556±447; AS 2,882±422; AS-GH 2,868±591 μm^2^; p=0.016). GH increased IGF-1 serum concentration (Sham 938±83; AS 866±116; AS-GH 1167±166 ng/mL; p<0.0001) and IGF-1 muscle protein expression and activated PI3K protein. Neural cell adhesion molecule (NCAM) immunofluorescence was increased in both AS groups. Catabolism-related intracellular pathways did not differ between groups.

**Conclusion:**

Short-term growth hormone attenuates left ventricular systolic dysfunction in rats with aortic stenosis-induced HF. Despite preserving body weight, increasing serum and muscular IGF-1 levels, and stimulating PI3K muscle expression, GH does not modulate soleus muscle trophism, satellite cells activation or intracellular pathways associated with muscle catabolism.

## INTRODUCTION

A major symptom in patients with chronic heart failure is a reduced tolerance to exercise caused by the early occurrence of dyspnea and fatigue. In addition to impaired ventricular function, noncardiac factors contribute to exercise intolerance. Metabolic, molecular, and functional skeletal muscle abnormalities have often been observed and are considered to play an important role in the symptoms [1]. Muscle wasting is a common disorder which often precedes cachexia and predicts frailty, poor quality of life, and mortality in heart failure [2, 3]. Despite being clinically important, its pathophysiology is not completely understood and a therapeutic approach for preventing muscle wasting and recovering muscle mass has not been established [3].

Growth hormone (GH) is essential for metabolic homeostasis and muscle growth and function [4]. GH exerts its effects either by direct action or mediated by the insulin-like growth factor-1 (IGF-1) [5, 6]. The GH/IGF-1 axis can suppress protein breakdown and stimulate skeletal muscle hypertrophy by inducing myocyte survival, differentiation, and proliferation [7-10]. However, the mechanisms involved in GH-induced mass preservation in pathologic conditions are not completely understood. IGF-1 can modulate muscle trophism by stimulating proliferation and differentiation of satellite cells, which are mononuclear and undifferentiated cells located between muscle fiber basal lamina and sarcolemma [11, 12]. At basal conditions, these cells remain in quiescent state; after harmful stimuli to the muscle, they are activated and named myogenic precursor cells or myoblasts [13, 14].

Clinical studies have shown that the GH/IGF-1 axis is impaired in heart failure with approximately 40% of patients presenting GH or IGF-1 deficiency [15-20]. IGF-1 is mainly reduced in patients with severe heart failure [21] or cardiac cachexia [17]. Low IGF-1 levels are associated with reduced skeletal muscle performance and poor outcome [21, 22]. Although GH administration produced beneficial effects on muscle strength in healthy men [23], its effects on cardiac remodeling have been poorly addressed. Results of therapeutic correction have been inconsistent, with some studies reporting unchanged functional cardiac indices [24], and others reporting improved left ventricular (LV) function, N-terminal prohormone brain natriuretic peptide (NT-proBNP) levels, and quality of life [19, 25].

Despite a potentially beneficial role on skeletal muscle, few authors have evaluated the effects of GH during heart failure [26]. In a previous study, we observed that GH preserved trophicity and attenuated interstitial fibrosis in aortic stenosis rat soleus muscle [27]. Also, GH prevented atrophy, apoptosis, and changes in myosin heavy chain in right heart failure [28, 29]. In this study, we tested the hypothesis that GH treatment improves muscle trophicity by activating satellite cells and the intracellular signaling pathways involved in muscular response to GH. Therefore, the main focus of this study was to evaluate the effects of GH administration on soleus muscle of rats with chronic heart failure induced by aortic stenosis. The effects of treatment on cardiac structures and left ventricular function were also assessed.

## RESULTS

### General characteristics of rats

In the AS group (n=13), 12 animals had tachypnea/labored respiration, 8 ascites, 7 pleuropericardial effusion, 6 thrombi in atria, 13 right ventricular hypertrophy, and 12 lung congestion. In AS-GH (n=10), 8 rats presented tachypnea/labored respiration, 4 ascites, 7 pleuropericardial effusion, 7 thrombi in atria, 6 right ventricular hypertrophy, and 8 lung congestion. The frequency of heart failure features did not differ statistically between groups. There was no evidence of heart failure in the Sham group (n=17). Anatomical data are shown in Table 1.

**Table 1:** Anatomical data

Variables	Sham (n=17)	AS (n=13)	AS-GH (n=10)
**BW (g)**	484 ± 42	415 ± 31*	476 ± 58#
**LV (g)**	0.84 ± 0.12	1.22 ± 0.26*	1.42 ± 0.24*
**RV (g)**	0.29 ± 0.06	0.50 ± 0.09*	0.60 ± 0.10*#
**Atria (g)**	0.09 ± 0.02	0.33 ± 0.10*	0.39 ± 0.06*
**Soleus (g)**	0.23 ± 0.03	0.19 ± 0.02*	0.21 ± 0.04
**LV/BW (mg/g)**	1.73 ± 0.17	2.93 ± 0.57*	2.91 ± 0.46*
**RV/BW (mg/g)**	0.61 ± 0.13	1.20 ± 0.22*	1.23 ± 0.19*
**Atria/BW (mg/g)**	0.20 ± 0.03	0.80 ± 0.23*	0.79 ± 0.11*
**Lung (g)**	2.17 ± 0.50	3.52 ± 0.76*	3.94 ± 1.18*
**Lung/BW (mg/g)**	4.53 ± 1.25	8.53 ± 1.96*	8.12 ± 2.74*

Data are expressed as mean ± standard deviation. AS: aortic stenosis; AS-GH: aortic stenosis treated with growth hormone; n: number of animals; BW: body weight; LV: left ventricle; RV: right ventricle. ANOVA and Tukey; * p<0.05 vs Sham; # p<0.05 vs AS.

### Echocardiographic evaluation

Before treatment, both AS groups had similar echocardiographic variables values, except for interventricular septum systolic thickness, which was higher in AS-GH than AS (Table 2). Final echocardiographic data are shown in Table 3. Due to technical problems, we could not perform final echocardiogram in two Sham rats and two AS-GH rats. Body weight was lower in AS than Sham and AS-GH. LV diastolic diameter-to-body weight ratio was higher in AS than Sham and AS-GH and did not differ between AS-GH and Sham. Diastolic and systolic posterior wall and interventricular septum thickness and left atria diameter were higher in both AS groups than Sham. Systolic interventricular septum thickness remained higher in AS-GH than AS. Endocardial fractional shortening was lower in AS than Sham and AS-GH, and midwall fractional shortening was lower in AS than Sham. Posterior wall shortening velocity, A-wave, E-wave deceleration time, and isovolumetric relaxation time were lower in both AS and AS-GH than Sham. E-wave and E/A ratio were higher in AS and AS-GH than Sham.

**Table 2:** Pre-treatment echocardiographic data

Variable	Sham (n=17)	AS (n=13)	AS-GH (n=10)
**BW (g)**	508.4 ± 41.1	451.7 ± 89.7	466.7 ± 59.5
**LVDD (mm)**	8.08 ± 0.45	8.51 ± 1.10	8.64 ± 0.82
**LVDD/BW (mm/kg)**	15.98 ± 1.21	19.29 ± 3.70*	18.69 ± 1.98*
**LVSD (mm)**	3.77 ± 0.47	4.07 ± 1.24	3.86 ± 0.90
**PWDT (mm)**	1.47 ± 0.09	2.25 ± 0.34*	2.46 ± 0.24*
**PWST (mm)**	2.98 ± 0.21	3.98 ± 0.34*	4.37 ± 0.72*
**IVSST (mm)**	2.67 ± 0.20	3.25 ± 0.24*	3.59 ± 0.27*^#^
**IVSDT (mm)**	1.48 ± 0.08	2.26 ± 0.34*	2.48 ± 0.26*
**AO (mm)**	3.80 ± 0.21	3.91 ± 0.27	3.93 ± 0.37
**LA (mm)**	4.99 ± 0.34	7.82 ± 1.08*	7.72 ± 0.87*
**LA/AO**	1.32 ± 0.15	2.00 ± 0.28*	1.97 ± 0.21*
**LA/BW (mm/kg)**	9.88 ± 1.07	17.79 ± 3.73*	16.68 ± 1.93*
**LVRWT**	0.35±0.03	0.52±0.11*	0.53±0.04*
**HR (bpm)**	287 ± 41	299 ± 34	299 ± 40
**EFS (%)**	53.4 ± 4.4	53.0 ± 9.5	55.5 ± 8.5
**MWFS (%)**	30.3 ± 3.1	28.7 ± 4.3	29.5 ± 3.9
**LVEF**	0.89 ± 0.03	0.89 ± 0.06	0.90 ± 0.05
**PWSV (mm/s)**	37.0 ± 2.5	27.8 ± 4.6*	28.7 ± 6.3*
**E-wave**	73 ± 7.0	141 ± 13.8*	122 ± 17.5*
**A-wave**	48 ± 6.7	34 ± 23.4*	25 ± 6.0*
**E/A**	1.58 ± 0.35	4.86 ± 1.31*	5.25 ± 2.03*
**EDT (ms)**	50 ± 6.9	33 ± 8.4*	34 ± 10.6*
**IVRT (ms)**	31±5.44	24±7.61*	21±6.32*
**IVRTn (ms)**	33 ± 4.4	23 ± 4.7*	19 ± 5.2*

Data are expressed as mean ± standard deviation. AS: aortic stenosis; AS-GH: aortic stenosis treated with growth hormone; n: number of animals; BW: body weight; LVDD and LVSD: left ventricle (LV) diastolic and systolic diameters, respectively; PWDT and PWST: LV posterior wall diastolic and systolic thicknesses, respectively; IVSST and IVSDT: interventricular septum systolic and diastolic thicknesses, respectively; AO: aortic diameter; LA: left atrial diameter; LVRWT: LV relative wall thickness; HR: heart rate (beats/min); EFS: endocardial fractional shortening; MWFS: midwall fractional shortening; LVEF: LV ejection fraction; PWSV: LV posterior wall shortening velocity; E/A: early-to-late diastolic mitral inflow ratio; EDT: E-wave deceleration time; IVRT: isovolumetric relaxation time; IVRTn: IVRT normalized to heart rate. ANOVA and Tukey; * p<0.05 vs Sham; # p<0.05 vs AS.

**Table 3:** Post-treatment echocardiographic data

Variables	Sham (n=15)	AS (n=13)	AS-GH (n=08)
**BW (g)**	484 ± 42	415 ± 31*	476 ± 58^#^
**LVDD (mm)**	7.73 ± 0.53	7.93 ± 1.42	7.98 ± 1.02
**LVDD/BW (mm/kg)**	15.54 ± 1.45	18.47 ± 3.20*	16.53 ± 3.62^#^
**LVSD (mm)**	3.16 ± 0.51	4.08 ± 1.74	3.20 ± 0.91
**PWDT (mm)**	1.43 ± 0.09	2.25 ± 0.25*	2.41 ± 0.37*
**PWST (mm)**	3.03 ± 0.21	3.79 ± 0.48*	4.33 ± 0.63*
**IVSST (mm)**	2.80 ± 0.19	3.05 ± 0.26*	3.44 ± 0.30*^#^
**IVSDT (mm)**	1.45 ± 0.08	2.26 ± 0.24*	2.40 ± 0.35*
**AO (mm)**	3.9 ± 0.1	4.1 ± 0.2*	4.0 ± 0.2
**LA (mm)**	4.9 ± 0.2	7.0 ± 0.8*	7.8 ± 1.2*
**LA/AO**	1.28 ± 0.06	1.74 ± 0.19*	1.92 ± 0.30*
**LA/BW (mm/kg)**	9.89 ± 0.79	16.22 ± 1.68*	15.87 ± 2.08*
**LVRWT**	0.37 ± 0.03	0.58 ± 0.10*	0.62 ± 0.16*
**HR (bpm)**	303 ± 43	298 ± 27	308 ± 21
**EFS (%)**	59.1 ± 5.6	49.8 ± 14.7*	59.8 ± 12.1^#^
**MWFS (%)**	32.8 ± 3.2	26.6 ± 7.9*	31.6 ± 6.3
**LVEF**	0.93 ± 0.03	0.87 ± 0.10	0.92 ± 0.08
**PWSV (mm/s)**	38.6 ± 7.2	25.2 ± 5.1*	25.6 ± 3.1*
**E-wave**	74 ± 8.5	132 ± 15.7*	121 ± 19.0*
**A-wave**	55 ± 15.9	26 ± 5.0*	23 ± 5.8*
**E/A**	1.45 ± 0.37	5.23 ± 0.97*	5.59 ± 1.31*
**EDT (ms)**	53 ± 4.3	33 ± 4.8*	29 ± 5.9*
**IVRT (ms)**	33 ± 4.18	24 ± 4.70*	21 ± 5.77*
**IVRTn (ms)**	34 ± 4.3	23 ± 5.0*	21 ± 5.5*

Data are expressed as mean ± standard deviation. AS: aortic stenosis; AS-GH: aortic stenosis treated with growth hormone; n: number of animals; ; LVDD and LVSD: left ventricle (LV) diastolic and systolic diameters, respectively; PWDT and PWST: LV posterior wall diastolic and systolic thicknesses, respectively; IVSST and IVSDT: interventricular septum systolic and diastolic thicknesses, respectively; AO: aortic diameter; LA: left atrial diameter; LVRWT: LV relative wall thickness; HR: heart rate (beats/min); EFS: endocardial fractional shortening; MWFS: midwall fractional shortening; LVEF: LV ejection fraction; PWSV: LV posterior wall shortening velocity; E/A: early-to-late diastolic mitral inflow ratio; EDT: E-wave deceleration time; IVRT: isovolumetric relaxation time; IVRTn: IVRT normalized to heart rate. ANOVA and Tukey; * p<0.05 vs Sham; # p<0.05 vs AS.

### Histologic analysis

**Figure 1:**
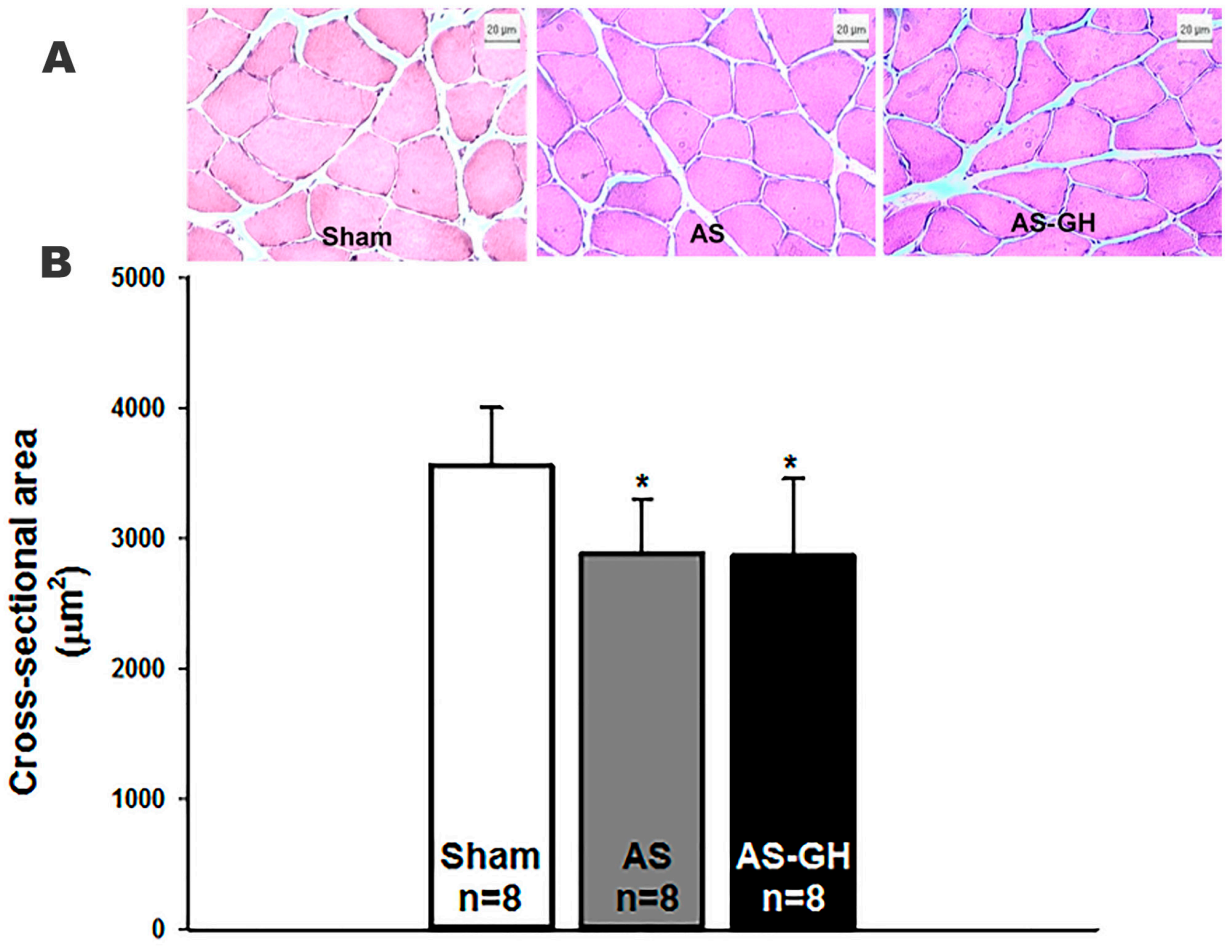
Photomicrographs of soleus muscle cross-sections stained with haematoxylin-eosin **(A)**. Soleus muscle cross-sectional area **(B)**. AS: aortic stenosis; AS-GH: aortic stenosis treated with growth hormone; n: number of animals. Data are expressed as mean ± standard deviation. ANOVA and Tukey; * p<0.05 vs Sham.

Figure 1A illustrates hematoxylin-eosin stained sections of soleus muscle. Soleus fiber cross-sectional area was smaller in both AS groups than Sham and did not differ between AS-GH and AS (Sham 3,556±447; AS 2,882±422; AS-GH 2,868±591 μm^2^; p=0.016 AS and AS-GH vs Sham; Figure 1B).

### Immunofluorescence

**Figure 2:**
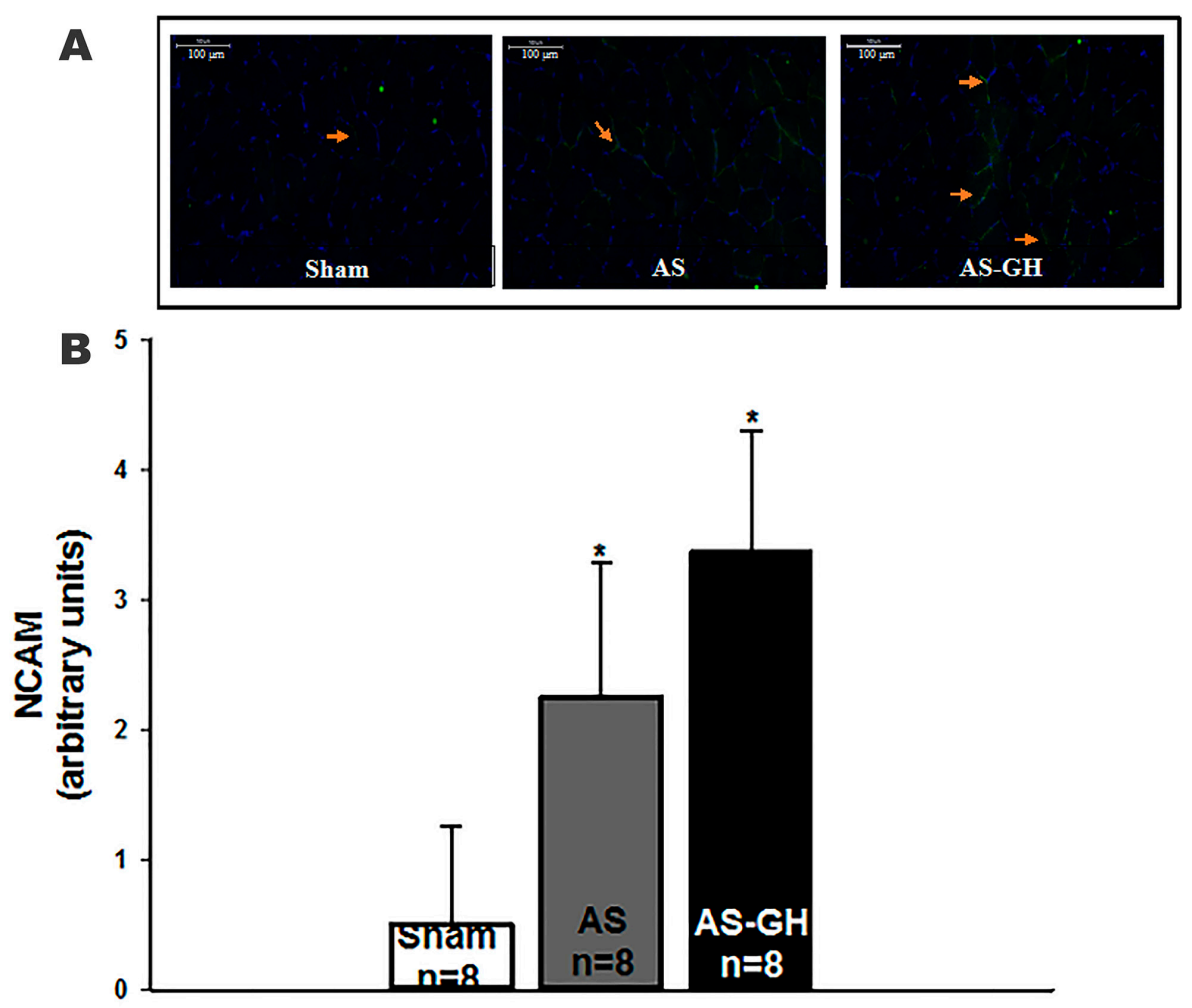
Representative immunofluorescence of soleus muscle cross-sections stained with anti-neural cell adhesion molecule (NCAM) and 4’,6-diamidino-2-phenylindole (DAPI) showing cell nucleus in blue and neural cell adhesion molecule (NCAM) in green **(A)**. Quantification of staining intensity of NCAM **(B)**. AS: aortic stenosis; AS-GH: aortic stenosis treated with growth hormone; n: number of animals. Data are expressed as mean ± standard deviation; ANOVA and Tukey; * p<0.05 vs Sham.

In soleus muscle, neural cell adhesion molecule (NCAM) staining was higher in both AS groups than Sham and did not differ between AS-GH and AS (Sham 0.50±0.76; AS 2.25±1.04; AS-GH 3.38±0.92 arbitrary units; p<0.001 AS and AS-GH vs Sham; Figure 2). MyoD [Sham 1.00 (0.00-2.00); AS 2.50 (2.00-3.00); AS-GH 2.50 (1.00-3.50) arbitrary units; p=0.14] and neonatal myosin heavy chain [Sham 0.00; AS 0.50 (0.00-1.00); AS-GH 0.50 (0.00-2.00) arbitrary units; p=0.19] staining did not differ between groups.

### Circulating IGF-1 levels

**Figure 3:**
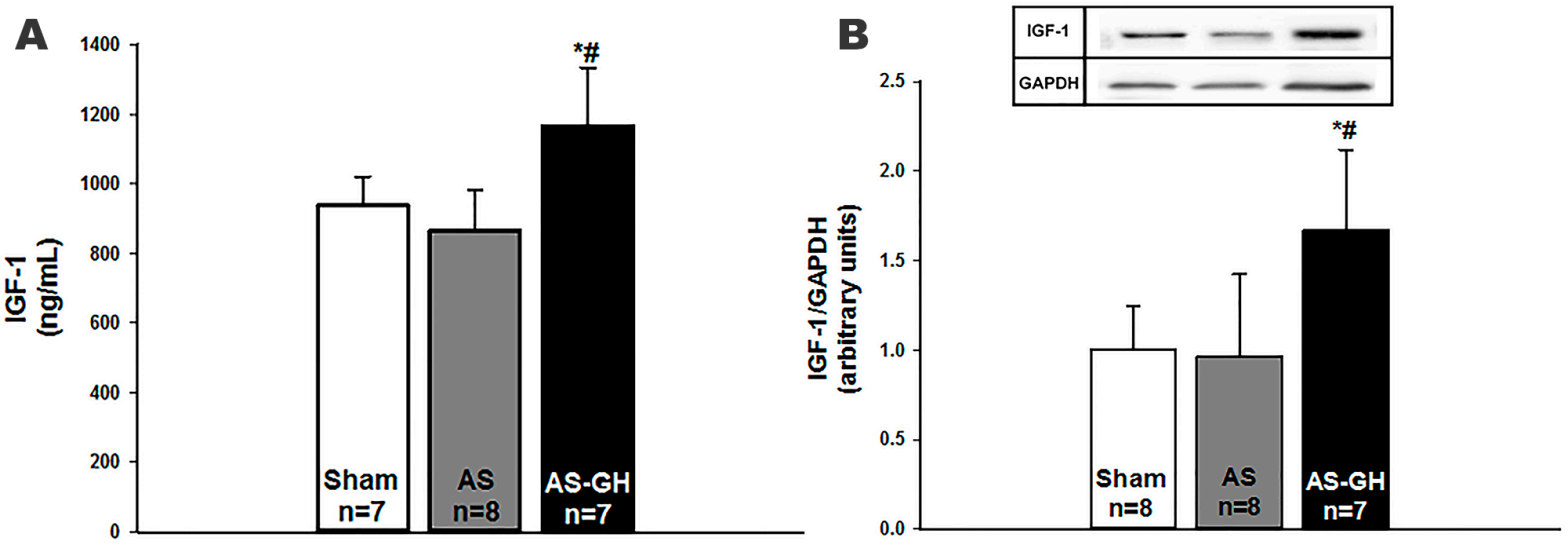
Insulin-like growth factor (IGF)-1: serum levels **(A)** and soleus muscle protein expression **(B)**. AS: aortic stenosis; AS-GH: aortic stenosis treated with growth hormone; n: number of animals. Data are expressed as mean ± standard deviation. ANOVA and Tukey; * p<0.05 vs Sham; # p<0.05 vs AS.

IGF-1 serum levels were higher in AS-GH than the other two groups (Sham 938±83; AS 866±116; AS-GH 1167±166 ng/mL; p<0.0001; Figure 3A).

### Protein expression

**Figure 4:**
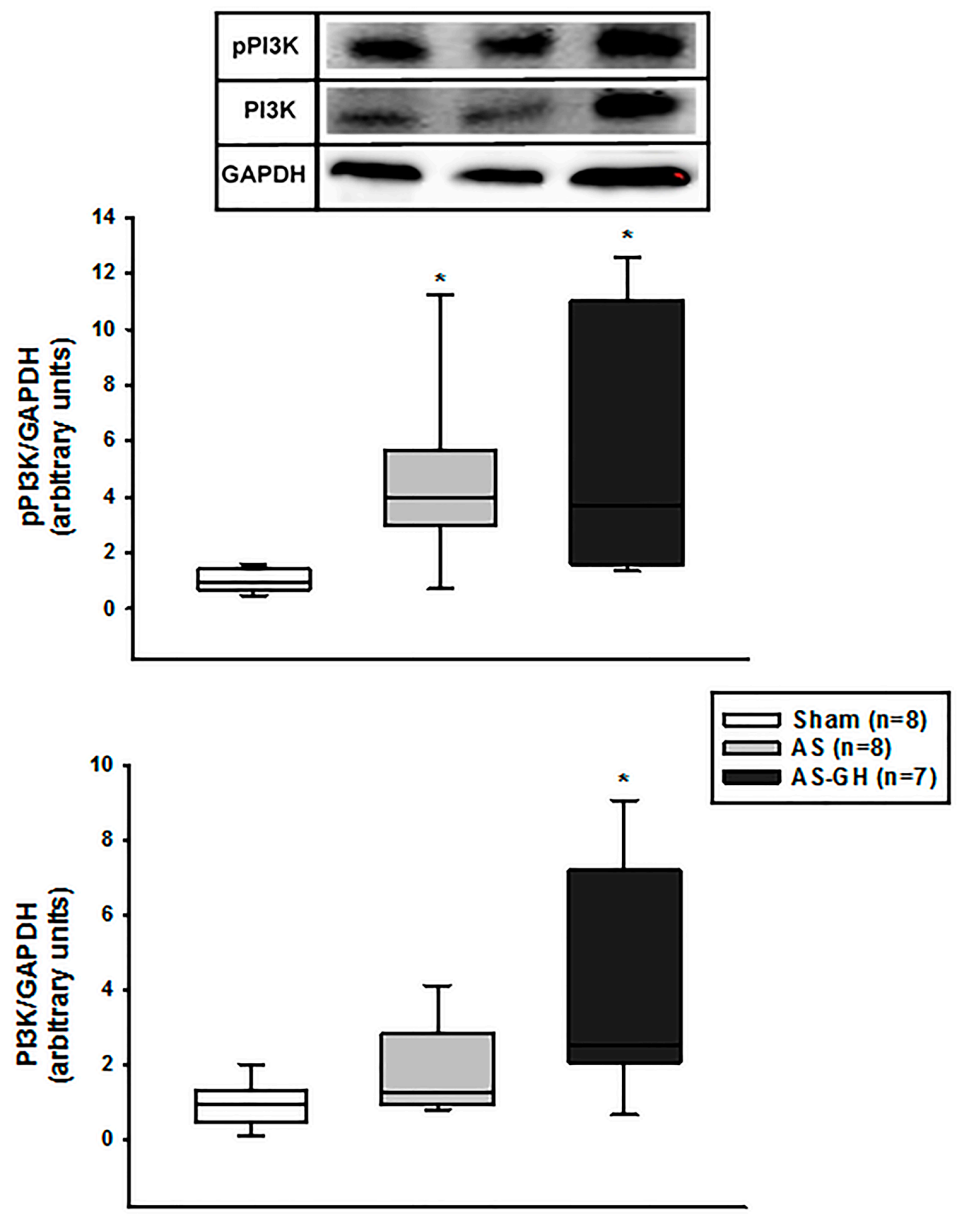
Phosphorylated phosphatidylinositol-3-kinase (PI3K) and total PI3K protein expression in soleus muscle assessed by Western blot AS: aortic stenosis; AS-GH: aortic stenosis treated with growth hormone; n: number of animals. Data are expressed as median and percentiles; Kruskal-Wallis and Student-Newman-Keuls; * p<0.05 vs Sham.

IGF-1 protein expression was higher in AS-GH than the other groups (Sham 1.00±0.25; AS 0.96±0.46; AS-GH 1.67±0.45 arbitrary units; p=0.004; Figure 3B). Phosphorylated-PI3K was higher in AS and AS-GH than Sham (Sham 0.94 (0.74-1.28); AS 3.99 (3.01-5.46); AS-GH 3.71 (1.97-9.62) arbitrary units; p= 0.004); total PI3K was higher in AS-GH than Sham (Sham 0.98 (0.63-1.32); AS 1.26 (1.00-2.51); AS-GH 2.51 (2.07-6.05) arbitrary units; p=0.037; Figure 4). The other proteins did not differ between groups (Table 4 and Supplementary Figure 1).

**Table 4:** Protein expression

Proteins (arbitrary units)	Sham (n=08)	AS (n=08)	AS-GH (n=07)
**MyoD**	1.04 (0.88-1.13)	1.10 (0.90-1.42)	1.47 (1.11-1.79)
**Myogenin**	1.00 ± 0.38	0.58 ± 0.36	0.87 ± 0.68
**MRF4**	0.88 (0.62-1.35)	0.60 (0.34-1.19)	1.33 (0.79-3.27)
**Akt**	1.00 ± 0.54	0.86 ± 0.35	0.86 ± 0.54
**Atrogin-1**	1.00 ± 0.44	0.73 ± 0.19	0.92 ± 0.18
**MuRF-1**	1.00 ± 0.90	0.53 ± 0.41	0.63 ± 0.23
**Myostatin**	1.00 ± 0.32	1.86 ± 1.09	2.05 ± 1.07
**Follistatin**	1.00 ± 1.11	1.42 ± 0.83	1.31 ± 1.44
**Pax-7**	1.00 ± 0.39	1.06 ± 0.63	0.74 ± 0.63

Data are expressed as mean ± standard deviation or median and percentiles. AS: aortic stenosis; AS-GH: aortic stenosis treated with growth hormone; n: number of animals. ANOVA and Tukey or Kruskal-Wallis and Student-Newman-Keuls; p>0.05 for all proteins.

## DISCUSSION

In this study, we evaluated the effects of growth hormone administration on satellite cell activation and intracellular signaling pathways related to skeletal muscle trophism in the soleus muscle of rats with aortic stenosis-induced heart failure.

Ascending aortic stenosis in rats is a useful model for studying pressure overload-induced heart failure. Three-to-four week-old rats have a clip placed around the ascending aorta. After clip placement, aorta diameter is preserved; as rats grow, stenosis progressively develops. The model has the advantage that, despite rapid onset of LV hypertrophy, LV dysfunction occurs slowly [30, 31], similar to that observed in human chronic pressure overload. Congestive heart failure is usually observed from 18 weeks after stenosis induction [27, 30, 32].

Before treatment, aortic stenosis rats presented left atrial dilation and LV hypertrophy with systolic and diastolic dysfunction. GH treatment attenuated systolic dysfunction as endocardial fractional shortening was higher in AS-GH than AS. Furthermore, midwall fractional shortening was lower in AS than Sham, but did not differ between AS-GH and Sham. The increased LV relative wall thickness in AS and AS-GH shows that both aortic stenosis groups had LV concentric hypertrophy. Heart failure was established by clinical and pathological features evaluated *in vivo* and *post mortem*. Lung weight and right ventricular hypertrophy are often used to diagnose heart failure in rats [33]. In this study, heart failure feature frequencies did not differ between AS-GH and AS groups. Therefore, despite attenuating systolic dysfunction, late GH administration did not modulate heart failure severity in aortic stenosis rats. The late treatment may have prevented differences in heart failure features between groups.

GH and IGF-1 play essential role in cardiac growth and performance [34]. Chronic administration of GH to normal rodents induces myocyte hypertrophy and improves cardiac performance [35]. However, in failing hearts, the effects of GH are still controversial [26, 32, 36-38]. We have previously observed that short-term GH treatment induces cardioprotection by attenuating myocardial fibrosis and systolic dysfunction in aortic stenosis rats [32]. In a small trial of heart failure patients with GH deficiency, GH replacement for 4 years improved peak oxygen consumption and systolic function with no major adverse events [25]. Therefore, our data reinforces the potential beneficial role of GH on cardiac remodeling and LV dysfunction.

In this study, GH preserved body weight. A similar result was reported post-myocardial infarction [26]. Cardiac cachexia is often observed in advanced stages of heart failure [39] and is associated with poor prognosis [40, 41]. Therefore, the effect of GH in preventing body mass loss may be important for heart failure prognosis. Although body weight preservation may suggest skeletal muscle preservation, this did not occur in soleus muscle. Atrophy of soleus muscle is a common finding in different experimental heart failure models [30, 42, 43].

As expected, GH increased both IGF-1 serum concentration and IGF-1 soleus protein expression. IGF-1 binding to its receptors activates several intracellular kinases. PI3K is a well-known signal transduction pathway activated by GH [44, 45]. Total PI3K expression was higher in AS-GH than Sham and phosphorylated-PI3K was higher in both AS groups than Sham. Akt/mTOR acts as IGF-1 downstream mediators [45]. Down-stimulation of Akt activates the forkhead box protein O (FoxO) family which increases atrogin-1 and MuRF-1, two potent inducers of protein degradation [7, 45-48]. It was therefore unexpected to observe that, despite activating IGF-1 and PI3K, GH failed to modulate atrogin-1 and MuRF-1 expression. Muscle atrophy is caused by the limitation of anabolic processes and/or activation of intracellular proteolytic systems [7]. In heart failure, proteolysis appears to overcome impaired protein synthesis [31, 54, 55]. We observed that the catabolic pathways evaluated in this study were not activated in rats with aortic stenosis-induced heart failure.

The myostatin/follistatin pathway has attracted increasing attention as a modulator of skeletal muscle mass [45, 49, 50]. Myostatin negatively regulates muscle size and follistatin acts as a myostatin antagonist. Balance between these two proteins is important in maintaining skeletal muscle mass [51]. Despite muscle atrophy in aortic stenosis rats, myostatin and follistatin expression did not differ between groups and was not changed by GH. In a previous study on infarcted rats, we observed that skeletal muscle atrophy was combined with preserved myostatin and reduced follistatin expression [43]. This study therefore showed that aortic stenosis-induced muscle atrophy is not associated with myostatin/follistatin changes.

Several molecular markers have been used to evaluate satellite cell activation; these include NCAM, MyoD, neonatal myosin heavy chain, and Pax-7 [13, 52, 53]. In this study, satellite cell activation was observed by increased NCAM staining in both the AS and AS-GH groups. Activation of quiescent satellite cells is related to cell proliferation and differentiation [13, 14]. We therefore showed that satellite cells are activated during heart failure and not modulated by GH administration.

Myogenic regulatory factors MyoD, myogenin, and MRF4 modulate the expression of several muscle proteins. In this study, their expression did not differ between groups. We have previously observed in aortic stenosis rats that GH increased soleus MyoD expression, preserved muscle trophicity, and attenuated interstitial fibrosis [27]. These rats however had a lower degree of cardiac injury as LV was not dilated and LV function was preserved except for a reduced LV posterior wall shortening velocity. It is therefore probable that GH produces beneficial effects in non-severe heart failure.

In summary, our results showed that late administration of GH, despite attenuating systolic dysfunction, failed to prevent/reverse soleus muscle atrophy. In fact, in disagreement with our hypothesis, GH did not modulate muscle trophism, satellite cell activation or the intracellular signaling pathways evaluated in this study. As GH preserved body weight, increased serum and muscular IGF-1 levels, and stimulated PI3K muscle expression, this study allowed us to raise two hypotheses: GH was initiated later when muscle atrophy was already established; or GH treatment was not long enough to reverse the muscle atrophy process. Therefore, although this study adds important information on the use of GH during heart failure, it was not possible to establish the best stage of heart failure to start GH in order to prevent/reverse skeletal muscle atrophy.

In conclusion, short-term growth hormone treatment attenuates left ventricular systolic dysfunction in rats with aortic stenosis-induced heart failure. Despite preserving body weight, increasing serum and muscular IGF-1 levels, and stimulating PI3K muscle expression, growth hormone does not modulate soleus muscle trophism, satellite cell activation, or the intracellular pathways associated with muscle catabolism.

## MATERIALS AND METHODS

### Experimental groups

Male Wistar rats (90-100 g) were purchased from the Central Animal House, Botucatu Medical School, Sao Paulo State University, UNESP, Botucatu, Brazil. Commercial chow and water were supplied *ad libitum*. Animals were housed in a room under temperature and light control in collective cages with four rats per cage. All experiments and procedures were approved by the Ethics Committee of Botucatu Medical School, Sao Paulo State University.

Aortic-stenosis was induced as previously described [56]. In summary, the animals were subjected to median thoracotomy after intramuscular anesthesia with ketamine hydrochloride (50 mg/kg) and xylazine hydrochloride (10 mg/kg). After dissecting the ascending aorta, a 0.6 mm stainless-steel clip was placed at approximately 3 mm from the aorta root. During surgery, the rats were manually ventilated using positive pressure and given 1 mL of warm saline solution intraperitoneally. Sham operated rats were used as controls.

We have previously observed that aortic stenosis rats start to present clinical heart insufficiency approximately 18 to 28 weeks after the surgery [27, 30, 57]. Therefore, beginning 18 weeks after surgery, rats were observed twice a week to detect heart failure signs, such as tachypnea and weight loss [58, 59]. After observing these features, rats were subjected to transthoracic echocardiogram to evaluate degree of cardiac injury, and randomly assigned to two groups: aortic stenosis with no treatment (AS), and aortic stenosis treated with GH (AS-GH). Rats were given daily subcutaneous injections of recombinant human growth hormone (2 mg/kg/day; Novo-Nordisk Laboratory, Bagsvaerd, Denmark) or vehicle for 14 days. Age-matched Sham rats were studied at comparable ages.

At the time of euthanasia, we evaluated the presence of anatomical heart failure features such as atria thrombi, ascites, pleuropericardial effusion, lung congestion (lung weight-to-body weight ratio > 2 standard deviations above Sham group mean), and right ventricular hypertrophy (right ventricle weight-to-body weight ratio greater than 0.8 mg/g) [60]. Aortic stenosis-induced cardiac remodeling was characterized by echocardiographic parameters. GH-induced increase in IGF-1 levels was determined by assessing its systemic concentration and muscular protein expression.

### Echocardiography

Echocardiographic evaluation was performed using a commercially available echocardiograph (General Electric Medical Systems, Vivid S6, Tirat Carmel, Israel) equipped with a 5-11.5 MHz multifrequency probe, as previously described [61-63]. Rats were anesthetized by intramuscular injection of a mixture of ketamine (50 mg/kg) and xylazine (0.5 mg/kg). A two-dimensional parasternal short-axis view of the LV was obtained at the level of the papillary muscles. M-mode tracings were obtained from short-axis views of the LV at or just below the tip of the mitral-valve leaflets, and at the level of the aortic valve and left atrium. M-mode images of the LV were printed on a black-and-white thermal printer (Sony UP-890MD) at a sweep speed of 100 mm/s. All LV structures were manually measured by the same observer (KO). Values obtained were the mean of at least five cardiac cycles on M-mode tracings. The following structural variables were measured: LV diastolic and systolic diameters (LVDD and LVSD, respectively), LV posterior wall diastolic and systolic thicknesses (PWDT and PWST, respectively), interventricular septum systolic and diastolic thicknesses (IVSST and IVSDT, respectively), left atrial diameter (LA), and aortic diameter (AO). LV relative wall thickness (LVRWT) was calculated as 2 X PWDT/LVDD. LV function was assessed by the following parameters: endocardial fractional shortening (EFS), midwall fractional shortening (MWFS), LV posterior wall shortening velocity (PWSV), early and late diastolic mitral inflow velocities (E and A waves, respectively), E/A ratio, E-wave deceleration time (EDT), isovolumetric relaxation time (IVRT), and IVRT normalized to heart rate (IVRTn).

### Morphological analysis

At euthanasia, rats were weighed and anesthetized with intraperitoneal sodium pentobarbital (50 mg/kg). Hearts were removed by thoracotomy and the atria and ventricles were separated and weighed. Soleus muscle was dissected, weighed, immediately frozen in liquid nitrogen, and stored at -80 °C.

Transverse sections approximately 8-10 μm thick of frozen soleus were cut in a cryostat at -20 °C and stained with hematoxylin and eosin. Muscle trophicity was assessed by measuring at least 200 cross-sectional fiber areas from each muscle [64]. Measurements were performed using a compound LEICA DM LS microscope attached to a computerized imaging analysis system (Media Cybernetics, Silver Spring, Maryland, USA). All analysis were performed by the same blind investigator.

### Immunofluorescence

We next evaluated the following molecular markers of satellite cell activation by immunofluorescence: NCAM, MyoD and neonatal myosin heavy chain. Soleus muscle transverse sections were fixed in 4% paraformaldehyde dissolved in phosphate-saline buffer (PBS), for 10 min at room temperature. Sections were then washed in PBS, blocked in PBS-albumin bovine serum (BSA) 5%-Triton X-100 for 10 min, and blocked again in PBS with 5% BSA for a further 20 min. Sections were incubated overnight in primary antibody (anti-NCAM, H-300 sc-10735; anti-MyoD, M-318 sc-760; and anti-neonatal myosin, N1.551 sc-53097; Santa Cruz Biotechnology, Santa Cruz, CA, USA) diluted in PBS, at 4 °C. Sections were then incubated with secondary antibody for one hour in a dark chamber at room temperature. Next, sections were washed in PBS, incubated with DAPI, and washed again in PBS. Coverslips were allocated using ProLong® Gold Antifade reagent (Molecular Probes® by Life Technologies). Sections were analyzed in fluorescence microscope (Olympus BX51, equipped with an Olympus U-RFL-T fluorescence emitter and Olympus DP72 camera, Panasonic). We evaluated 10 histologic frames in each section. Immunofluorescence reaction was analyzed for staining intensity according to pre-established scores: +++ strong, ++ moderate, + weak or - without staining.

### Western blotting

Soleus protein levels were analyzed by Western blot according to a previously described method [65, 66]. Protein expression of myogenic regulatory factors (MyoD, M-318 sc-760; myogenin, M-225 sc-576e; MRF4, Myf-6 C-19 sc-301), IGF-1 (H-70 sc-9013), atrophy pathway related-proteins (PI3K, PI 3-kinase p85α B-9 sc-1637; p-PI3K, p-PI 3-kinase p85α (Tyr 467) sc-293115; Akt, Akt1 G-5 sc-55523; atrogin-1, MAFbx H-300 sc-33782; MuRF-1, H-145 sc-32920; myostatin, GDF-8 N-19-R sc-6885-R; and follistatin H-114 sc-30194), and Pax-7 (Pax-3/7 H-208 sc-25409; Santa Cruz Biotechnology, Santa Cruz, CA, USA) was evaluated. Protein levels were normalized to GAPDH (6C5 sc-32233, Santa Cruz Biotechnology). Samples were separated on polyacrylamide gel and then transferred to a nitrocellulose membrane. After blockade, the membrane was incubated with primary antibodies overnight at 4 °C. The membrane was then washed with PBS and Tween 20 and incubated with secondary peroxidase-conjugated antibody for 90 min at room temperature. ECL Western Blotting Substrate (Pierce Protein Research Products, Rockford, USA) was used to detect bound antibodies. The membrane was then stripped (Restore Western Blot Stripping Buffer, Pierce Protein Research Products, Rockford, USA) to remove previous antibody. After blockade, the membrane was incubated with anti-GAPDH antibody [67, 68].

### Circulating IGF-1 levels

IGF-1 serum concentration was assessed by enzyme linked immunosorbent assay (ELISA) using Quantikine® ELISA Mouse/Rat IGF-I kit (R&D Systems, Minneapolis, MN, USA). The procedure was performed according to manufacturer instructions.

### Statistical analysis

Data are expressed as mean ± standard deviation or median and percentiles. Comparisons between groups were performed by one-way analysis of variance (ANOVA) followed by the Tukey test for parametric data, or by Kruskal-Wallis test followed by Student-Newman-Keuls for non-parametric data. Heart failure feature frequencies were analyzed by the Goodman’s test. The level of significance was set at 5%.

## SUPPLEMENTARY MATERIALS FIGURE


