# Supplementary material for: Effects of growth hormone on cardiac remodeling and soleus muscle in rats with aortic stenosis-induced heart failure
Source: Oncotarget. 2017 Aug 24;8(47):83009–21. doi: 10.18632/oncotarget.20583 (PMC5669945; doi:10.18632/oncotarget.20583)
Supplement: Supplementary file 1 [file oncotarget-08-83009-s001.pdf]

Effects of growth hormone on cardiac remodeling and soleus muscle in rats with aortic stenosis-induced heart failure

SUPPLEMENTARY MATERIALS

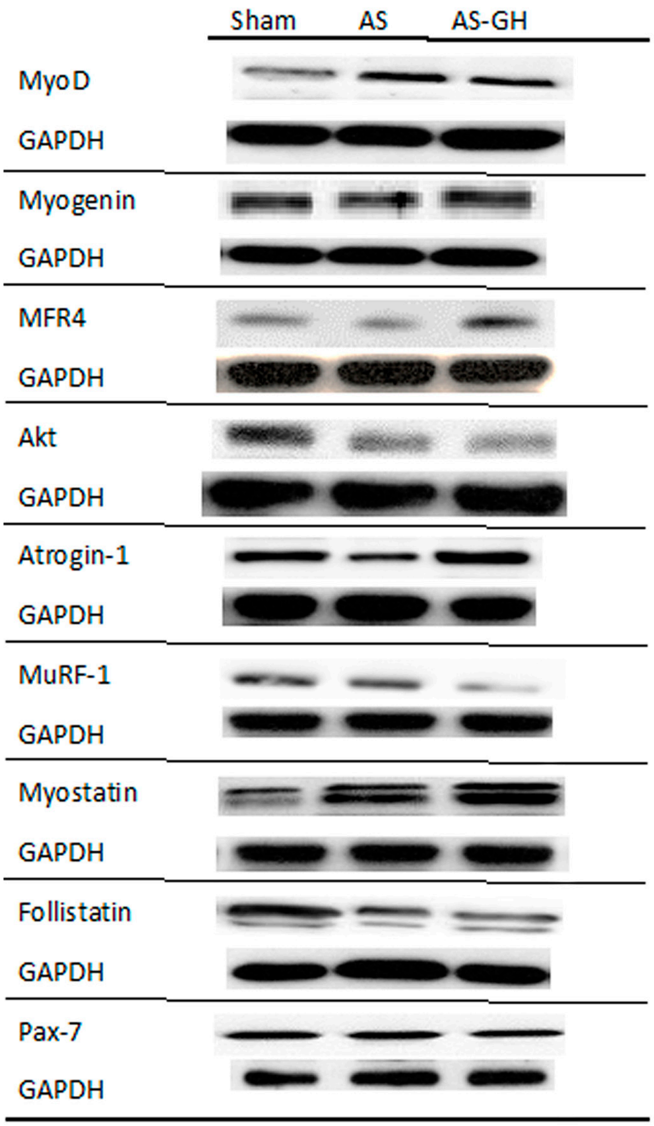

Supplementary Figure 1: Representative immunoblots of soleus muscle proteins analyzed by Western blot. Quantitative results are shown in Table 4. AS: aortic stenosis; AS-GH: aortic stenosis treated with growth hormone.
